# Effect of light at different wavelengths on polyol synthesis of silver nanocubes

**DOI:** 10.1038/s41598-022-23959-3

**Published:** 2022-11-10

**Authors:** Rasoul Gheitaran, Abbas Afkhami, Tayyebeh Madrakian

**Affiliations:** 1 Faculty of Chemistry, Bu-Ali Sina University, Hamedan, Iran; 2 D-8 International University, Hamedan, Iran

**Keywords:** Chemistry, Materials chemistry

## Abstract

Despite the presence of light-sensitive species in the polyol synthesis of silver nanocubes, the influence of light on it has yet to be investigated. Herein, we demonstrated that light radiation, by generating plasmon-based hot electrons and subsequently increasing the reduction rate of Ag^+^ in the system, in addition to enhancing the growth rate of nanocubes, causes twinned seeds, which these seeds are then converted into nanorods and right bipyramids. With shorter, higher energy wavelengths, Ag^+^ reduction progresses more quickly, resulting in structures with more twin planes. The overlap of the excitation wavelength and the band gap of Ag_2_S clusters formed in the early stages of synthesis accelerates the rate of reaction at low-energy excitation. According to our findings, the surfactant polyvinylpyrrolidone acts as a photochemical relay to drive the growth of silver nanoparticles. Overall, this work emphasizes the impact of excitation light on polyol synthesis as a technique for generating Ag nanocubes of various sizes.

## Introduction

The unique and remarkable physicochemical properties of noble-metal nanostructures in comparison with their respective bulk materials have led to many researchers being interested in these nanomaterials and exploiting them in a diversity of applications^1–6^. Ag nanocrystals received great interest because of their utilities in varied applications, including localized surface plasmon resonance (LSPR), surface-enhanced Raman scattering (SERS), catalysis, optical labeling, imaging, biosensing, bactericidal, and medicine^7–22^.

The physical and chemical attributes of Ag nanomaterials are based on their size, shape, and composition. Therefore, in the last few decades, researchers have tried to synthesize Ag nanoparticles with controlled size and shapes, such as spheres, right bipyramids^23,24^, tetrahedrons^25^, octahedrons^26^, decahedrons^27^, cubes^19,28–34^, bars^20^, rods/wires^35–37^, and plate-like shapes^38–41^, among others.

Among these synthetic shapes, Ag nanocubes, owing to their potential performance as a votive template for the synthesis of nanoboxes, nanocages, and nanoframes of several metals, including Au, Pt, Pd^42–45^ and, as superb substrates for surface-enhanced Raman scattering (SERS) sensing and also as a superior catalyst for epoxidation reactions, have obtained a lot of attention^46,47^.

Because of the above-mentioned explanations, it is important to produce Ag nanocubes with a well-controlled size and perform an investigation of parameters that can be effective on their size and reproducibility.

Up to now, polyol reduction is the most extensively used method for generating Ag nanocubes was reported for the first time in 2002 by Sun and Xia^48^. Refinements of this synthetic approach of Ag nanocubes using many additives either individually or simultaneously, such as HCl/NaCl^49–51^, NaSH/Na_2_S^52^, Fe^2+^/Fe^3+^ salts^36^, and Br^−^ ion^29^ for access to well-defined nanocubes, have been described. Also, Xia et al.^33^ were able to improve this synthesis method by replacing silver precursor from AgNO_3_ to CF_3_COOAg. Over the past decade, myriad studies have been conducted to investigate the role of these additives. Indeed, S^2−^ species by the formation of Ag_2_S clusters act as heterogeneous nucleant and Fe^2+^/Fe^3+^ compounds take part as oxidative etching agents.

More recently, it has been demonstrated that Cl^−^ and Br^−^ play a role in directing cube shape via thermodynamic control, and polyvinylpyrrolidone (PVP) as a capping agent assists in producing Ag nanocubes by kinetic control^53^. Although this method is light sensitive due to the presence of species such as Ag_2_S, AgCl, and Ag nanoparticles, few studies have been conducted to date to investigate the effect of light on polyol reduction methods. After a comprehensive study of the literature, we found just two reports that only investigated the effect of sunlight on these polyol-based systems. They both used protocols that were not reproducible. Ashkarran et al.^54^, inspected the impact of solar light on this approach, in which the silver precursor is reduced by heated ethylene glycol in the presence of PVP, and a trace amount of Na_2_S. According to Xia’s group results^30^, the reproducibility of this procedure is low, so the results obtained from their work cannot be generalized/validated. This is because aside from light, many parameters may affect the systems that cannot be controlled. In the other work, Im et al.^55^ claimed that the light from fluorescent tubes or the sun can affect the reproducibility of synthesis by its impact on the formed AgCl during the reaction. They deduced AgCl photodecomposed to Ag and afterward the Ag atoms sit act as heterogeneous nucleation places therewith yielding irregularly shaped Ag nanoparticles. Recently, Rioux et al.^49^ investigated the role of Cl^-^ in the polyol synthesis of Ag nanocubes. They discovered that Ag nanocubes are produced even when the polyol process is run under light conditions and without stirring, implying that AgCl is unlikely to serve as a heterogeneous nucleant for Ag nanocube synthesis.

As mentioned previously, the effect of light has not been studied on polyol-based synthetic methods at different irradiation wavelengths. Here, in this paper, we explore how light affects the polyol synthesis of Ag nanocubes during the entire reaction. It was found that using light at different wavelengths affects both the size and the shape of the Ag nanocrystals.

## Materials and methods

### Chemicals and materials

Ethylene glycol (EG, ≥ 99.5% lot no. K48128121), silver trifluoroacetate (CF_3_COOAg, ≥ 99.7%), sodium sulfide hydrate (Na_2_S∙*x*H_2_O), aqueous hydrochloric acid solution (HCl, 37%), sodium borohydride (NaBH_4_) and silver nitrate (AgNO_3_) were all obtained from Merck Company. Poly (vinyl pyrrolidone) (PVP, MW **≈** 55,000) was purchased from Sigma-Aldrich. Deionized (DI) water with a resistivity of 18.2 MΩ cm was used throughout the experiment.

### Synthesis of Ag nanocubes in EG

The recipe for silver nanocube synthesis was modified based on Xia’s paper^33^. In a typical experiment, 5 mL EG was poured into a 100 mL round bottom flask and loosely closed, then heated under magnetic stirring at a stirring rate of 700 rpm in an oil bath set to 150 °C. After 1 h, 60 µL of 3 mM Na_2_S in EG solution was injected. Two minutes after the addition of Na_2_S, 0.5 mL of 3 mM HCl solution was injected into the reaction solution, followed by the addition of 1.25 mL of 20 mg/mL PVP in EG. After another 2 min, 0.4 mL of 282 mM CF_3_COOAg in EG was injected into the mixture. Except for the addition of chemicals, the flask was closed with glass stoppers during the entire process, following the original Xia experimental method. Using glass pipets, aliquots were obtained at different stages of synthesis and immediately injected into 1.5 mL centrifuge tubes filled with cool acetone. After 1.5 h, the growth was stopped by immersing in an ice-water bath. To remove the residual EG and PVP, all of the samples were centrifuged and washed with acetone and DI water. The final product was dispersed in DI water. To investigate the effect of light and excitation wavelength on the polyol synthesis, the reaction solution was irradiated with (1) a 200-W incandescent lamp, (2) light-emitting diodes (LEDs) of different excitation wavelengths as the exciting source in three wavelengths 465, 528 and 628 nm with intensities of approximately 0.06, 0.24, and 0.06 W respectively. The reaction flask was located 10 cm away from the light output window. Also in dark condition, the flask was wrapped with aluminum foil.

### Silver nanoparticles without PVP

Were prepared using procedures described in the literature^56,57^. In brief, 10 mL silver nitrate solution (1 mM) was added drop by drop to 30 mL of 2 mM NaBH_4_ under magnetic stirring at 0 °C until the solution became bright yellow. Before aggregation of Ag nanoparticles, their open-circuit voltage was recorded. To modify the Ag nanocrystals with PVP, 5 mg PVP was added into 20 mL of the same bare Ag nanoparticles under vigorous stirring and left for 20 min.

### Electrochemistry

Electrochemical studies were carried out using a three-electrode electrochemical cell, in which a platinum rod served as the counter electrode and a gold wire functioned as the reference electrode. A working silver nanoparticle/FTO electrode with a 2 cm^2^ area was employed. The supporting electrolyte contained 0.1 M KNO_3_ with 10% (vol%) ethylene glycol (tuned to pH ≈ 4 via HCl addition to mimic the synthesis pH conditions). To make Ag nanocrystal photoelectrodes, Ag nanoparticles were drop-cast onto a fluorine-doped tin oxide (FTO) glass substrate and then heated at 70 °C for 30 min to assure enough adhesion with the underlying substrate. For bare FTO in solution with 10% (vol%) ethylene glycol and 0.1 M potassium nitrate, no photovoltage change was detected. Also, no photovoltage was detected in alkaline conditions or in the absence of ethylene glycol.

### Instrumentation

Scanning electron microscopy (SEM) images were taken using a field-emission microscope (FESEM; TESCAN MIRA3 LMU, Czech Republic) operated at an accelerating voltage of 3–20 kV. Transmission electron microscopy (TEM) images were captured using a Philipps EM 208S microscope operated at 100 kV. Extinction spectra of all the Ag nanocrystals were obtained using a mini-WPA UV–vis Spectrophotometer. Chronopotentiometry measurements with zero net current (open circuit) were performed using an Autolab PGSTAT 302 N model potentiostat/galvanostat (Eco-Chemie, Netherlands) that was controlled by NOVA 1.11 software. The homemade LED source was made of nine LEDs (3 × 3) screwed into an aluminum plate that helped the heat transfer to a heat sink attached. The LEDs emission spectra are available in the Supplementary Information.

## Results and discussion

### Synthesis of Ag nanocubes in room light and dark conditions

**Figure 1:**
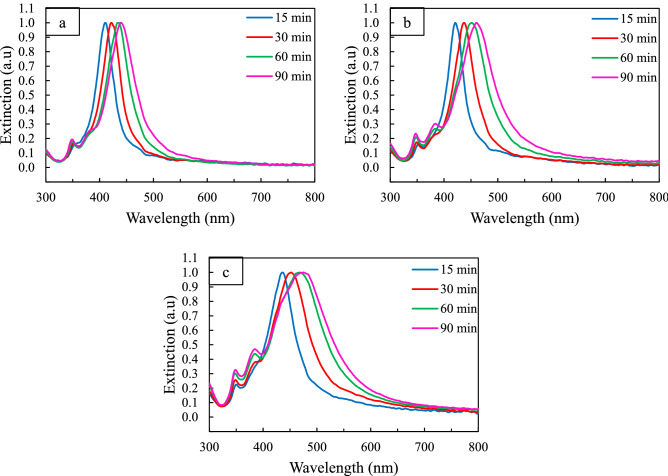
UV–Vis spectra of Ag nanoparticles aliquots collected at different reaction times in different light condition (**a**) dark, (**b**) room light, (**c**) 200-W incandescent lamp.

In the first step, to make sure light is an effective parameter in the synthesis of Ag nanocubes, synthesis was carried out in two different light conditions, while other effective parameters in the reaction were held constant: room light (under the fluorescent lamp) and dark (to cover the reaction vessel with aluminum foil). Then the reaction was probed over time. The changes in the morphology of Ag nanocubes were monitored by UV–Vis spectroscopy and Scanning Electron Microscopy (SEM). Figure 1 shows normalized UV−Vis extinction spectra of the obtained Ag nanoparticles during a reaction from 15 to 90 min at certain intervals. According to the literature, the presence of a shoulder peak at about 355 nm indicates the formation of Ag nanocubes^29,31^. As the reaction progresses, a shoulder peak around 380 nm appears, indicating the growth of corners and edges as well as the transformation of nanocubes from a truncated form to sharp corners^58^. These two shoulder peaks are observed in the extinction spectra of both conditions, but it should be noted that the 380 nm shoulder peak appears earlier under light than in dark conditions. In addition, more changes in the position of the main LSPR peak relative to time, under light irradiation conditions, indicate faster growth kinetics under light conditions (Table S1).

Therefore, it can be concluded that by changing the light intensity, we can synthesize Ag nanocubes of different sizes. Xia et al.^31^ used diethylene glycol instead of ethylene glycol to achieve smaller nanocubes, while in our method, by removing light from the system, the reduction rate of the reaction can be reduced and small nanocubes can be easily synthesized. On the other hand, up to now, many seed-mediated methods have been developed for the synthesis of nanocubes with larger sizes, which seems to be achieved by using excitation light with an intensity and a specific wavelength. To prove this claim, a 200-W incandescent lamp that could provide more light intensity (Fig. S9) was used as the light source. As shown in Fig. 1c, the λ_max_ variations of extinction spectra over time show more changes than the previous two conditions (Fig. 1a, b). These observations confirm the increase in reaction kinetics in the presence of light (Table S1).

**Figure 2:**
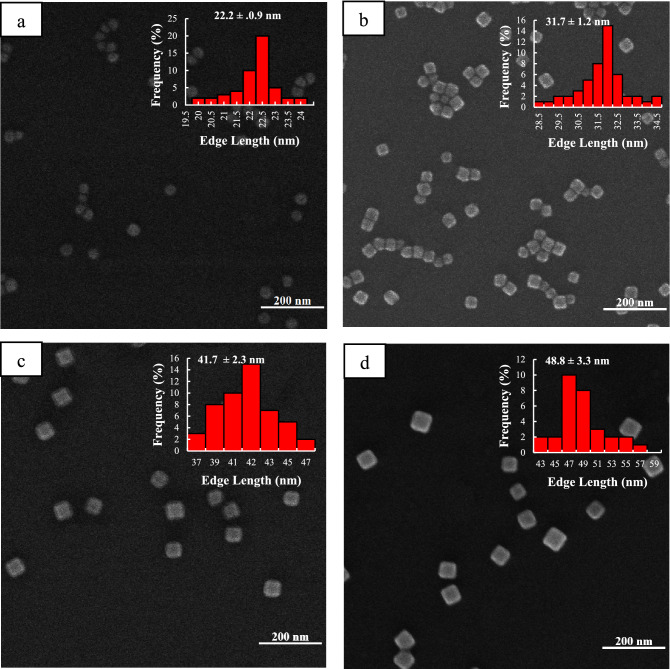
SEM images of Ag nanocubes obtained under dark conditions at different reaction times: (**a**) 15, (**b**) 30, (**c**) 60, and (**d**) 90 min. The insets show the size distribution of the Ag nanocubes.

**Figure 3:**
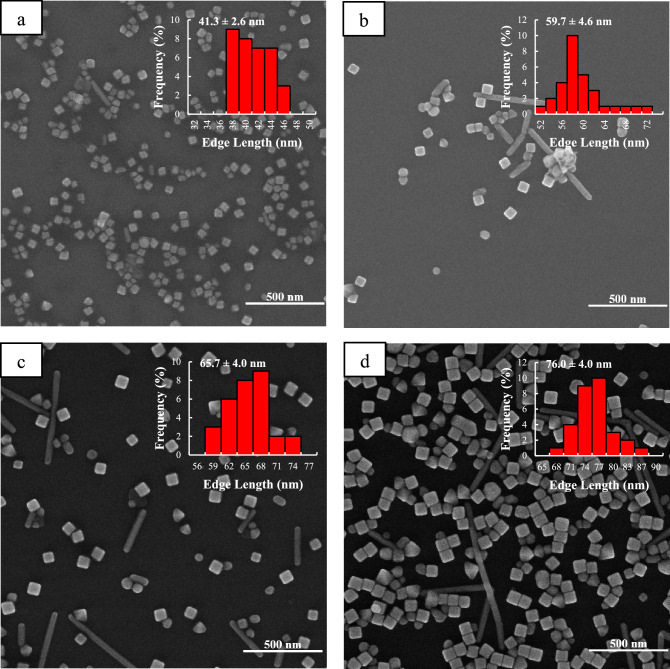
SEM images of Ag nanocubes obtained under irradiance with 200-W incandescent lamp at different reaction times: (**a**) 15, (**b**) 30, (**c**) 60, and (**d**) 90 min. The insets show the size distribution of the Ag nanocubes.

In addition, more structural information about nanoparticles can be obtained using these extinction spectra. To achieve this, the obtained spectra were fitted using the Lorentz function. As shown in Fig. S1, 15 min after the addition of CF_3_COOAg, only the dipole plasmon modes of the Ag nanocubes^58^ are present when synthesis is performed under dark conditions, while in another two conditions, in addition to these plasmon modes, there are several others that overlap with the original ones. The peaks at 419 and 505 nm can be related to the presence of pseudo-spherical particles and longitudinal plasmon of silver nanorods, respectively in Fig. S1e^59,60^. Also, the extinction spectra obtained under the three above conditions were studied at 90 min after the addition of CF_3_COOAg (Fig. S1). As shown in Fig. S1b, in the dark conditions, only dipolar plasmons related to Ag nanocubes are observed, while in the other two conditions (Figs. S1d, S1f), in addition to these peaks, there are other peaks that overlap with the main ones. Xia et al.^23^ established that right bipyramids with the same edge length as Ag nanocubes show a red-shift about 50 nm, hence the two peaks in positions 497 and 512 could be related to the presence of right bipyramids, and also the 647 and 757 nm could represent the longitudinal plasmon of silver nanorods in both room light and 200-W lamps, respectively^59,60^. The images of SEM taken from the Ag nanocubes at different time points of the reaction well confirm the aforementioned notes (Figs. 2, 3, S2).

Figure 2 shows SEM images of the synthesized Ag nanocubes that were obtained at different times in dark conditions. The edge length of Ag nanocubes increases from 22 to 48 nm while being portrayed as just a cube shape in the images, but in the other two conditions, in addition to Ag nanocubes, a small number of right bipyramids and nanorods are observed. Since the changes in light intensity are in order of dark < room light < lamp 200 watts, the varying of the edge length of nanocubes changes in the same way (Figs. 2, 3, S2). This observation is compatible with our studies on extinction spectra.

**Figure 4:**
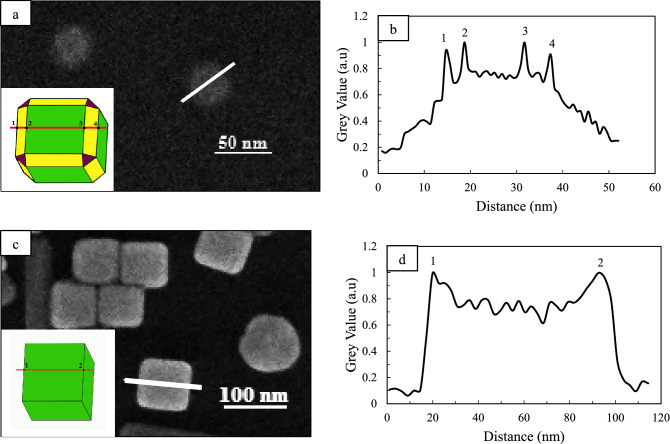
High-magnification SEM images of Ag nanocubes obtained in dark condition at 15 min (**a**), and in the presence of 200-W incandescent lamp (**c**). The insets are the scheme of Ag nanocubes obtained in different conditions. (**b**, **d**) grey value intensity along the white line in (**a**, **c**) respectively, the intensity of the edges are very different from the background noise and flat points thus edges on the line can be identified.

It is known that contrast in SEM images arises from the Secondary Electron (SE) efficiency difference for the different incident angles of the primary electrons that radiate to the specimen surface. Therefore, the contrast changes of SEM images depend on the topography of the specimen surface, so that in places on the surface that are in the form of peaks or ridges, the contrast is high and in places where the surface is flat or valley, the contrast is lower, respectively^61^. The sharpness of the edge and corners of nanocubes were investigated using grey value analysis of SEM images. As much as the slope of the marked areas with the red circles in Fig. S3a increases results in sharper edges of the Ag nanocubes. As shown in Fig. S3, over time, the sharpness of the edges and corners increase in all three cases, but as mentioned earlier, due to the increase in growth rate owing to the higher light intensity in the presence of a 200-W lamp, the sharpness of the edges and corners is also higher. This observation is compatible with our studies on extinction spectra. This method was also used to show that the synthetic nanoparticles are edge-truncated cubes at 15 min under dark conditions (Fig. 4a, b). At the four points marked in Fig. 4b, the contrast of the image is higher than that at the other points due to the edges, and it is observed as four peaks in the grey analysis plot. Also, Fig. 4c and d show the complete formation of the edges of nanocubes as two sharp peaks in the gray analysis plot (two points 1 and 2 in Fig. 4d).

## Synthesis of Ag nanocubes in the presence of excitation light with different wavelengths

**Figure 5:**
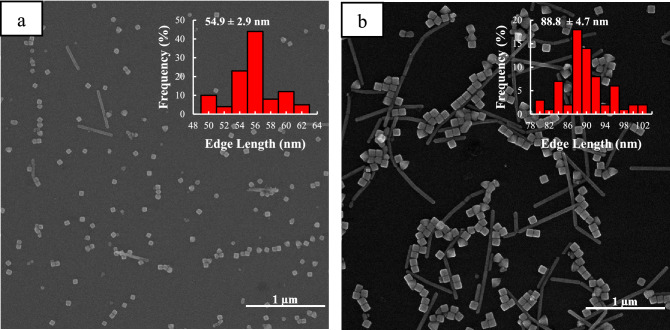
SEM images of Ag nanoparticles obtained under irradiance with excitation wavelength of 465 nm at different reaction times, (**a**) 15 min, (**b**) 90 min. The insets show the size distribution of the Ag nanocubes.

It has been proven in the literature that plasmon-based synthesis of Ag nanoparticles is affected by excitation wavelengths of the incident light^38,40,41^. Therefore, to examine the effect of excitation light on the polyol synthesis of Ag nanocubes more rigorously, the synthesis was evaluated at three different wavelengths (465, 528, and 628 nm) with an identical light intensity. At 465 nm excitation, it was observed from the SEM images (Fig. 5) and extinction spectra (Fig. S4), the reaction rate is much higher than that for previous modes (Table S2), and in addition to the nanocubes, which are the main product of the reaction, nanorods and a smaller amount of right bipyramids are also seen in final products.

As can be seen in the SEM image (Fig. 5a) 15 min after adding the silver source, in addition to nanocubes, there are some nanoparticles with pseudo-spherical shapes in the system, which according to the SEM image at 90 min (Fig. 5b), it turns out that most of these seed particles eventually evolve into nanorods and right bipyramids. SEM images also show that their structure was multiply twinned and planar-twinned, respectively. In this synthetic method, sulfide species were added to form Ag_2_S clusters that act as nucleation sites in the system. On the other hand, Xia et al.^62^ showed when the reaction kinetics in seed-based syntheses increases, the two reduction pathways consisting of solution reduction and surface reduction occur simultaneously. This allows homogeneous nucleation to happen in the system which causes the formation of small nanoparticles in the system at the same time as the seeds grow. Consequently, we hypothesize that the presence of these pseudo-spherical particles could be owing to the enhanced rate of reduction in the reaction. According to the literature plasmon-based syntheses of Ag nanoparticles, such as prisms, pyramids, and rods, are critically dependent on both the wavelength of incident light and reduction kinetics^24,37,38,41^.

Mirkin et al., showed that at shorter wavelengths, higher energies (400–450 nm), due to a greater rate of Ag^+^ reduction, multiply twinned seeds in the reaction solution would be the dominant product. They explained that due to the high overlap of shorter excitation wavelengths with the LSPR of small seeds formed in the early stages of synthesis, the rate of silver reduction is also higher than that in other conditions^28^.

The direct relationship between light intensity and nanoparticles growth rate indicates that synthesis is a plasmon-mediated reaction. So, since the intensity of incident light at the wavelength of 528 nm could be adjusted in a wide range, we performed the synthesis in two modes of light intensity, equal to two other wavelengths, and also in conditions where the intensity was about four times.

**Figure 6:**
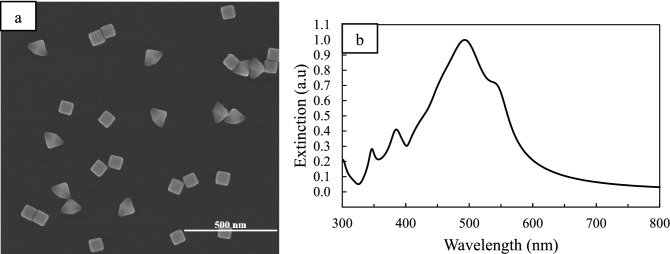
(**a**) SEM image of Ag nanoparticles obtained under irradiance with LED source at 528 nm after 90 min while light intensity is ¼ of normal mode. (**b**) UV–vis spectrum of Ag nanoparticles shown in (**a**).

When synthesis is performed under light intensity conditions equal to the other two modes, it is clear from the extinction spectra (Figs. S4a, 6b) that the particle size variation is less than that when synthesis is carried out at 465 nm. In addition, a new peak is observed at a distance of about 50 nm from the original peak (Fig. 6b), which, as shown in the image of SEM (Fig. 6a), this peak can be related to the right bipyramids, which account for about 40% of synthesis products.

It has previously been proven that in the presence of excitation light of 500–550 nm, the predominant product of the synthesis will be the right bipyramids^24,28^. The light intensity of the excitation wavelength at 528 nm was increased up to 4 times. It was observed that the trend of changes in the extinction spectrum and particle size approaches the 465 nm irradiation (Figs. 5, S5, S4a,S4b, and Table S2). The TEM image obtained from nanoparticles (Fig. 8) shows that the nanorods have a pentagonal cross-section as well as right bipyramids are as truncated-corners, and Ag nanocubes, which are the main product of the reaction (70%), have sharp edges and corners.

The synthesis was very interesting in red light conditions (628 nm). We expected it to have little effect on synthesis due to lower energy and lower spectral overlap, but as shown in Fig. S6b, in addition to nanocubes, nanorods and a smaller number of right bipyramids are still present in synthesis. Zheng et al.^37^ showed that in the presence of incident light (600–750 nm), seeds with a pentagonal twin structure are eventually converted to rods. Since the Ag_2_S clusters formed in the early stages of synthesis are light-sensitive^63^, the optical band-gap of these clusters was calculated from their absorbance spectrum (Fig. S7a) and found that it has a high spectral overlap with the excitation wavelength at 628 nm (Fig. S7b). Here, it accelerates the reduction rate in the seed formation step and causes multiple twinned seeds to form in the system, eventually growing into nanorods in the presence of red light. It is worth noting that in terms of aspect ratio, the nanorods obtained are larger than those gained in the previous two wavelengths, which may be because, under the conditions of red light, longitudinal plasmons are activated, causing the growth at the tip of the nanorods and the increase of the aspect ratio.^37^ Also, considering the LSPR spectra, it is clear that the edge length changes of nanocubes are less than the other two wavelengths (Fig. S4, and Table S2).

It was determined that the wavelength and intensity of the incident light affect the synthesis, but the question is, how? The effect of light on nanoparticle synthesis has been researched by several groups. All of them suggested that electron–hole pairs created by Landau damping during the optical excitation of surface plasmons on metal nanoparticles were responsible for these photochemical processes^40,64–66^. Due to the incompatibility of timescales between the hot-electron lifespan (~ fs-ps)^67,68^ and the slow kinetics of Ag^+^ reduction (~ µs-ms)^69–71^, it is improbable that these electrons may directly reduce the Ag precursors. Hence, in this plasmon-driven photocatalytic model, the presence of an intermediary chemical species bound on the Ag nanoparticle surface is essential to extend the hot-electron lifetime and accelerate Ag^+^ reduction.

**Scheme 1:**
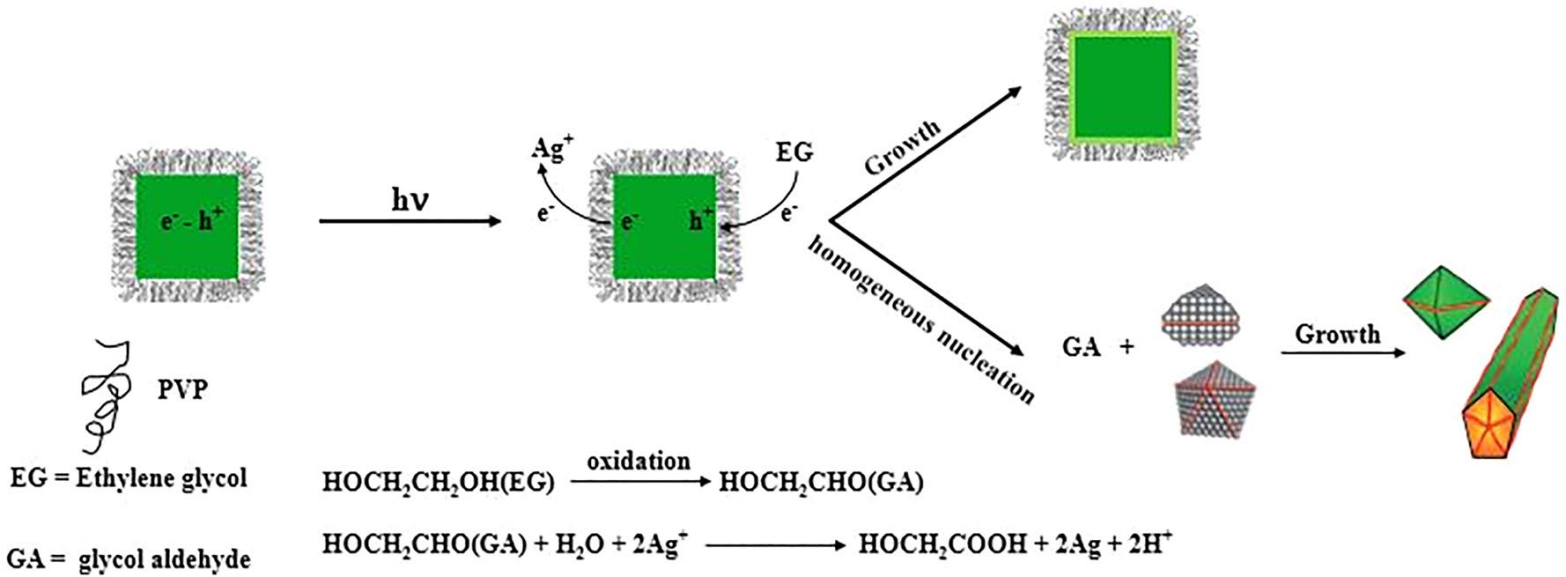
Schematic illustration of redox reaction occurring on the Ag nanocubes surface upon plasmon excitation in the presence of PVP that acts as a surfactant and hot electron stabilizer.

On the other hand, it has previously been proven that surfactant PVP at acidic pH induces a positive surface charge on the surface of nanoparticles, and thus plasmon-generated hot electrons are coulombically stabilized over long timescales at this created PVP/Ag junction, and also hot holes are swiftly scavenged by hole scavengers (such as methanol)^72^. Therefore, considering that PVP exists in our synthetic system in identical conditions to the ones described above (acidic conditions due to the addition of HCl to the reaction media). It is reasonable to expect it to serve as a hot electron stabilizer. On the other hand, ethylene glycol, which functions as the solvent in the system, can operate as a hot hole scavenger and contribute to the growth kinetics of Ag nanoparticles by oxidizing to glycolaldehyde (reducing agent in our reaction)^73^. As a result, increasing the concentration of glycolaldehyde in the solution might result in homogenous nucleation (Scheme 1). Monitoring the open-circuit potential (OCP) of Ag nanocrystal electrodes under visible-light irradiation provided electrochemical evidence for the direct participation of PVP in the plasmon-driven development of Ag nanoparticles^74–76^.

**Figure 7:**
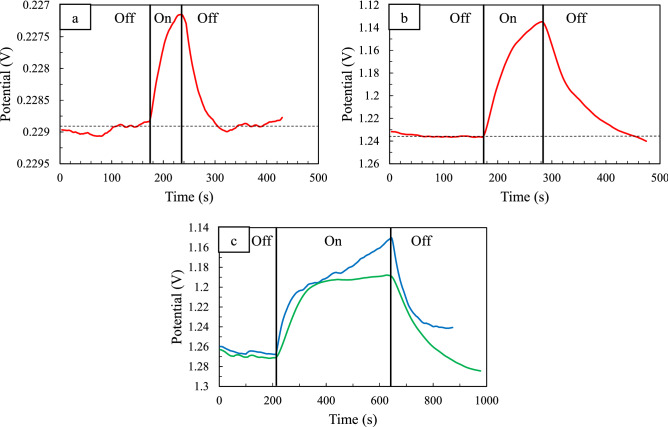
Plot of open circuit potential vs time for the same Ag nanoparticles photoelectrode without (**a**) and with (**b**) the modification of PVP during light irradiation (*λ*_inc_ = 465 nm). (**c**) Plot of open circuit potential vs time for Ag nanoparticles (*λ*_max_ = 430 nm) photoelectrode under irradiance at 465 nm (blue) and 528 nm (green) with equal light intensity. Horizontal dashed lines indicate open-circuit potential (OCP) baselines taken in the dark conditions. The terms on and off refer to the sample under irradiation (on) and the sample in the dark (off).

Accordingly, photovoltage under open-circuit conditions was measured using chronopotentiometry. Figure 7a and b show that in the presence of incoming light with a wavelength of 465 nm, the photovoltage of PVP-stabilized Ag nanoparticles is significantly higher than that of ones without this capping agent (~ 100 mV vs. 2 mV). Also, the effect of excitation wavelength overlap with the extinction spectrum of Ag nanoparticles on photovoltage is depicted in Fig. 7c. As can be seen, the photovoltage recorded at higher spectral overlap (465 nm) is greater than its value at 528 nm. Taken together, these findings show that PVP as a surfactant aids in the build-up of hot electrons on Ag nanoparticles over lengthy timescales, allowing Ag precursors to be reduced. According to the aforementioned findings, the physical position of PVP on the surface of nanoparticles has a significant role in the anisotropic growth of Ag nanocrystals.

**Figure 8:**
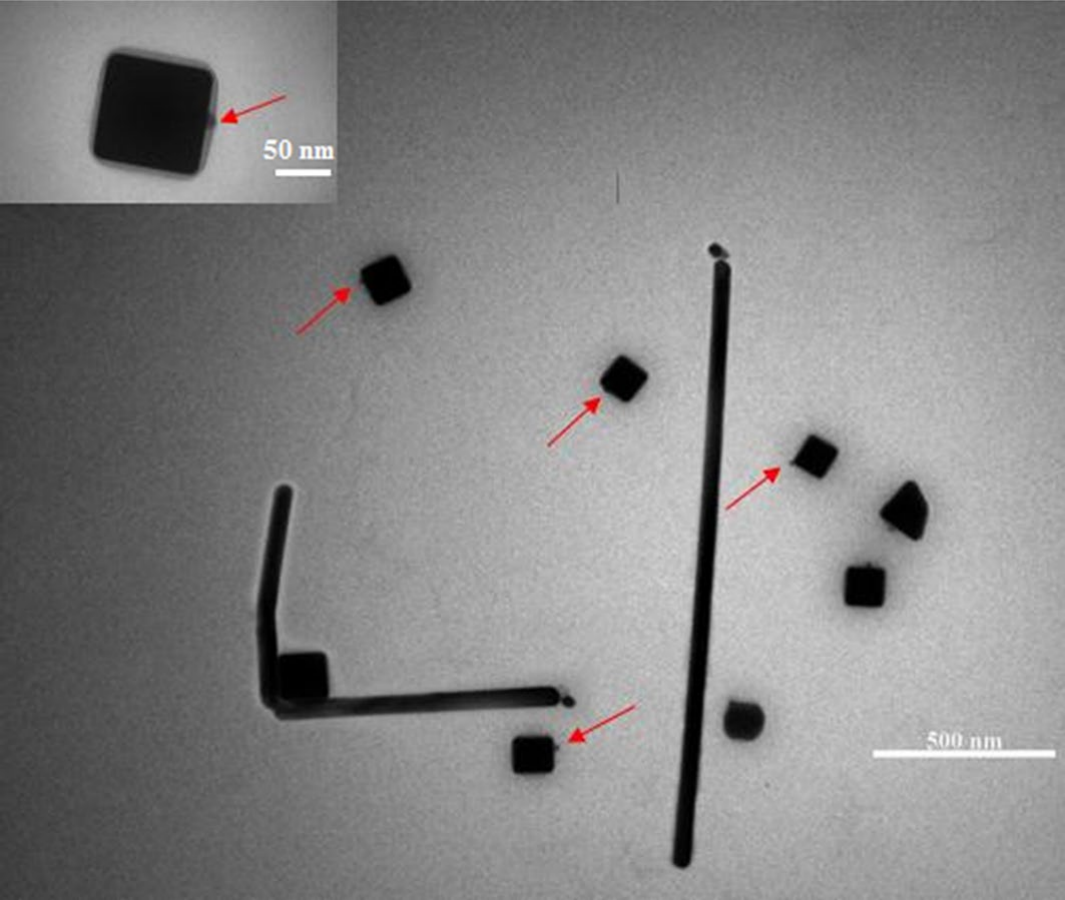
Low-magnification and high magnification (inset) TEM images of Ag nanoparticles obtained under irradiance with LED source at 528 nm after 90 min. Red arrows show that silver is deposited randomly on all facets.

It has already been demonstrated that PVP coverage on Ag (100) facets is twice as great as on Ag (111) facets by calculating the surface density of PVP on different facets of Ag nanoparticles^53^. As a result, the deposition speed of silver is higher on the (111) facets and nanocubes are formed due to kinetic control. Therefore, we speculate that in the presence of radiant light, the growth rate of (111) facets is further reinforced, and this leads to the earlier appearance of large-sized Ag nanocubes in the reaction media and following the production of Ag nanocubes with (100) facets, due to the uniformity of PVP coating on all facets, silver is deposited randomly on all of them (Fig. 8).

## Conclusion

In conclusion, we have revealed that by controlling the incident light and modulating the wavelength, we were able to synthesize a wide range of Ag nanocubes in terms of size: in the absence of light, uniform nanocubes with small and tunable sizes, and with application of light, still adjustable nanocubes in size, but with larger dimensions. It was found that incident light, in combination with polyol synthesis, accelerates the rate of silver reduction and the formation of nanocubes in the polyol system. Ag^+^ reduction proceeds more quickly with shorter, higher energy wavelengths, resulting in structures with more twin planes and, following that, nanorods and right bipyramids are formed in the system. It was also demonstrated that at low energy excitation, the rate of Ag^+^ reduction is accelerated due to the overlap of the excitation wavelength and the band gap of Ag_2_S clusters generated in the early stages of synthesis, resulting in the presence of nanorods and right bipyramids in the synthesis. According to the open-circuit potential (OCP) results, the surfactant PVP acts as a photochemical relay to drive the growth of anisotropic Ag nanoparticles from Ag seeds generated in the early stages of the polyol system. Overall, this study highlights the influence of incident light on polyol synthesis as a strategy for producing Ag nanocubes in a wide range of sizes.

## Supplementary Information

Supplementary Information 1.

Supplementary Information 2.

## Data Availability

The datasets used and/or analyzed during the current study and supporting the conclusions of this article are included in this article. These datasets are also available from the corresponding author upon reasonable request.
