# Supplementary material for: Effect of light at different wavelengths on polyol synthesis of silver nanocubes
Source: Sci Rep. 2022 Nov 10;12:19202. doi: 10.1038/s41598-022-23959-3 (PMC9649587; doi:10.1038/s41598-022-23959-3)
Supplement: Supplementary file 1 — Supplementary Information 1. [file 41598_2022_23959_MOESM1_ESM.docx]

**Supplementary** **Information**

**Effect of light at different wavelengths on polyol synthesis of silver nanocubes**

*Rasoul Gheitaran^1^, Abbas Afkhami^1,2,*^, Tayyebeh Madrakian^1^*

^1^Faculty of Chemistry, Bu-Ali Sina University, Hamedan, Iran

^2^ D-8 International University, Hamedan, Iran

**Table of Contents**

Table S1. Wavelength (λ) of the major LSPR peak of Ag nanocubes at different reaction S3

times in different light conditions: dark, room light, and 200-watt incandescent lamp

Figure S1. Fitted UV-Vis spectra of Ag nanoparticles obtained in different light conditions S4

Figure S2. SEM images of Ag nanocubes obtained under room light conditions S5

Figure S3. Grey value analysis of SEM images S6

Figure S4. UV-Vis spectra of Ag nanoparticles obtained under irradiance with three S7

different wavelength

Table S2. Wavelength (λ) of the major LSPR peak of Ag nanocubes obtained under S8

irradiance with three different wavelengths at different reaction times

Figure S5. SEM images of Ag nanoparticles obtained under irradiance with the excitation S9

wavelength of 528 nm

Figure S6. SEM images of Ag nanoparticles obtained under irradiance with excitation S10

wavelength of 628 nm

Figure S7. Uv-vis spectrum of the reaction solution, 15 seconds after adding the silver S11

source and Plot of (αhν)^2^ vs. (hν)

Figure S8. The emission spectra of LEDs S12

Figure S9. The emission spectra of incandescent and fluorescent lamps S13

| Time(min.)  Condition | 15 | 30 | 60 | 90 |
| --- | --- | --- | --- | --- |
| dark | **410 nm** | **422 nm** | **435 nm** | **440 nm** |
| Room light | **421 nm** | **437 nm** | **452 nm** | **460 nm** |
| 200-watt incandescent lamp | **436 nm** | **452 nm** | **468 nm** | **475 nm** |

**Table S1.** Wavelength (λ) of the major LSPR peak of Ag nanocubes at different reaction times in different light conditions: dark, room light, and 200-watt incandescent lamp.

b

a

d

c

f

e

**Figure S1.** Fitted UV-Vis spectra of Ag nanoparticles obtained in different light conditions, (a,b) dark, (c,d) room light, (e,f) 200-watt incandescent lamp, and (a,c,e) 15 min, (b,d,f) 90 min. The spectra were fitted with the Lorentz function using Fityk software.

b

a








d

c








**Figure S2.** SEM images of Ag nanocubes obtained under room light conditions at different reaction times: (a) 15, (b) 30, (c) 60, and (d) 90 min. The insets show the size distribution of the Ag nanocubes.

a


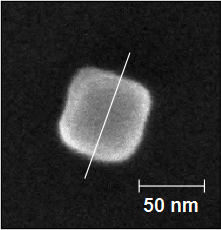


b

A

d

c

**Figure S3.** Grey value analysis of SEM images. (a) An example of how to obtain gray value intensity changes. The grey value intensity along the white line in the inset SEM image was obtained using AxioVision Software and it is plotted in terms of distance. The other three plots show grey value analysis of SEM images of Ag nanocubes obtained at different time points 15 min (blue), 30 min (red), 60 min (green), and 90 min (pink) under three different conditions: (b) dark, (c) room light, (d) 200-watt incandescent lamp.

b

a

c

**Figure S4.** UV-Vis spectra of Ag nanoparticles obtained under irradiance with three different wavelengths (a) 465, (b) 528, (c) 628 nm at different reaction times. The light intensity at 528 nm was four times that of the other two wavelengths.

| Time(min.)  Condition | 15 | 30 | 60 | 90 |
| --- | --- | --- | --- | --- |
| Blue light (465 nm) | **458 nm** | **485 nm** | **527 nm** | **541 nm** |
| Green light (528 nm) | **434 nm** | **450 nm** | **473 nm** | **493 nm** |
| Red light (628 nm) | **428 nm** | **444 nm** | **466 nm** | **483 nm** |

**Table S2.** Wavelength (λ) of the major LSPR peak of Ag nanocubes obtained under irradiance with three different wavelengths at different reaction times and equal light intensity.

a





b





**Figure S5.** SEM images of Ag nanoparticles obtained under irradiance with an excitation wavelength of 528 nm at different reaction times, (a) 15 min, (b) 90 min. The light intensity at 528 nm was four times that of the other two wavelengths. The insets show the size distribution of the Ag nanocubes.

a





b





**Figure S6.** SEM images of Ag nanoparticles obtained under irradiance with an excitation wavelength of 628 nm at different reaction times, (a) 15 min, (b) 90 min. The insets show the size distribution of the Ag nanocubes.

a

b

**Figure S7.** (a) Uv-vis spectrum of the reaction solution, 15 seconds after adding the silver source. At this moment, the reaction solution is a mixture of Ag_2_S clusters and a minimal amount of silver nanoparticles. (b) The plot of (αhν)^2^ vs. (hν). According to this plot, a direct transition occurs at 2 eV (≈ 620 nm)

b

a

**λ_max_ = 528 nm**

**FWHM = 35 nm**

**λ_max_ = 465 nm**

**FWHM = 50 nm**

**λ_max_ = 628 nm**

**FWHM = 35 nm**

c

**Figure S8.** The emission spectra of (a) blue, (b) green, and (c) red LEDs.

**Figure S9.** The emission spectra of incandescent and fluorescent lamps. The light intensity of incandescent and fluorescent lamps was 0.2 and 0.001 W, respectively.
