# Supplementary material for: Effect of light at different wavelengths on polyol synthesis of silver nanocubes
Source: Sci Rep. 2022 Nov 10;12:19202. doi: 10.1038/s41598-022-23959-3 (PMC9649587; doi:10.1038/s41598-022-23959-3)
Supplement: Supplementary file 2 — Supplementary Information 2. [file 41598_2022_23959_MOESM2_ESM.docx]

**Supplementary Information**

Fitted UV-Vis spectra and SEM images of Ag nanoparticles obtained in different light conditions, Grey value analysis of SEM images, Uv-vis spectrum of the reaction solution at 15 s and Plot of (αhν)^2^ vs. (hν), emission spectra of LEDs, emission spectra of incandescent and fluorescent lamps.
